# Impact on Survival on Interval between Surgery and Adjuvant Chemotherapy in Completely Resected Stage IB-IIIA Lung Cancer

**DOI:** 10.1371/journal.pone.0163809

**Published:** 2016-11-18

**Authors:** Bing-Yen Wang, Jing-Yang Huang, Wei-Heng Hung, Ching-Hsiung Lin, Sheng-Hao Lin, Yung-Po Liaw, Jiunn-Liang Ko

**Affiliations:** 1 Division of Thoracic Surgery, Department of Surgery, Changhua Christian Hospital and Chung Shan Medical University, Taichung, Taiwan; 2 Department of Public Health and Institute of Public Health, Chung Shan Medical University, Taichung City 40201, Taiwan; 3 School of Medicine, Kaohsiung Medical University, Kaohsiung, Taiwan; 4 Institute of Genomics and Bioinformatics, National Chung Hsing University, Taichung, Taiwan; 5 Department of Family and Community Medicine, Chung Shan Medical University Hospial, Taichung 40201, Taiwan; 6 Division of Chest Medicine, Department of Internal Medicine, Changhua Christian Hospital, Changhua, and Chung Shan Medical University, Taichung, Taiwan; 7 Department of respiratory care, College of health sciences, Chang Jung Christian University, Tainan, Taiwan; 8 Institute of Medicine, Chung Shan Medical University, Taichung, Taiwan; 9 Department of Medical Oncology and Chest Medicine, Chung Shan Medical University Hospital, Taichung, Taiwan; University of North Carolina at Chapel Hill School of Medicine, UNITED STATES

## Abstract

**Background and Objectives:**

Complete surgical resection is recommended for early stage lung cancer, and adjuvant chemotherapy is given for stage IB to IIIA disease. No studies have examined the best timing to administer chemotherapy after surgery in lung cancer. This study was to investigate the optimal timing of adjuvant chemotherapy after surgical resection.

**Methods:**

Data collected from the Taiwan National Health Insurance Research Database between January, 2004 and December, 2010 were retrospectively analyzed. Patients with stage IB to IIIA lung cancer underwent complete surgical resection and adjuvant chemotherapy were included. A total of 1522 patients were included. The patients were divided into 4 groups according to the interval between surgery and chemotherapy: group 1, < 30 days; group 2, 30–45 days; group 3, 46–60 days; group 4 > 60 days. Univariate and multivariate regression analyses were used to identify prognostic factors for overall survival.

**Results:**

The numbers of patients in groups 1, 2, 3, and 4 were 153, 161, 290, and 818, respectively. The 5-year survival rate was 41% in group 1, 48% in group 2, 50% in group 3, and 35% in group 4 (p<0.001). The median survival time was 44.50 months in group 1, 59.53 months in group 2, 67.33 months in group 3 and 36.33 months in group 4 (p<0.001) Survival rate is the poorest when chemotherapy is delayed beyond 60 days after surgical resection Multivariate analysis also indicated the interval between surgery and first course of chemotherapy more than 60 days after surgery was an independent risk factor for survival.

**Conclusions:**

Timing of chemotherapy after surgery is associated with poorer survival in lung cancer patients.

## Introduction

An estimated 1.8 million new lung cancer cases are diagnosed every year, accounting for about 13% of total cancer diagnoses [1], and an estimated about 1.6 million patients died of lung cancer every year worldwide. Lung cancer was also the leading cause of cancer-related death in Taiwan [2]. Although medical knowledge and surgical techniques have advanced, the long-term survival of patients with lung cancer is still poor. The high mortality rate is primarily because most patients are diagnosed at an advanced stage, and the response rate to chemotherapy is not satisfactory.

According to current guidelines published by the American Society of Clinical Oncology and the National Comprehensive Cancer Network (NCCN), complete surgical resection and mediastinal lymph node dissection provides an opportunity to cure lung cancer, and improve long-term survival in patient with stage I or a subset of stage II (T1-2, N1) disease. Multimodality therapy is suggested for most patients with stage III disease. Systemic therapies, such as chemotherapy or targeted therapies, are recommended for patients with stage IIIB and stage IV disease. When patients receive complete surgical resection, observation is recommended for patients with stage IA disease, and for those with stage IB to IIIA disease adjuvant chemotherapy is suggested after complete surgical resection to decrease the distant recurrent rate and improve survival.

In spite of undergoing complete curative surgical resection, a certain percentage patients still died within 5 years [3]. The main cause of death of these patients after surgical resection is distant recurrence. Thus, study of adjuvant chemotherapy after surgery has focused on eradicating micrometastasis. Several randomized trials have shown that adjuvant chemotherapy after complete surgical resection has a survival benefit for patients with lung cancer before stage IIIA [4–7]. Some studies, however, have reported that adjuvant chemotherapy before stage IIA disease is not necessary after surgical resection [8–10].

Current literature, however, has not examined the best timing to administer adjuvant chemotherapy for patients with stage IB-IIIA after complete surgical resection. In other cancers, such as breast cancer or colorectal cancer, several studies have examined the timing of adjuvant chemotherapy after surgery [11–19]. These studies have suggested that a delay in administering adjuvant chemotherapy may worsen overall survival.

The purpose of our study was to investigate the influence of the interval between surgery and administration of adjuvant chemotherapy on overall survival in patients with lung cancer. Therefore, we performed a national database analysis to compare the long-term survival of patients with lung cancer who received surgery and adjuvant chemotherapy based on the interval between surgery and first course of adjuvant chemotherapy. This is the first study which has examined the influence of the interval between surgery and adjuvant chemotherapy in patients with lung cancer.

## Materials and Methods

### Database

The Taiwan National Health Insurance Research Database (NHIRD) is the largest, most complete and most detailed database in Taiwan, and records clinical information about individuals diagnosed with lung cancer. In Taiwan, the majority of the population must join National Health Insurance, and the NHIRD records clinical health information of approximately 98% of Taiwan’s 23 million people. It contains chief demographic and diagnostic information, along with 1 principal and up to 4 secondary International Classification of Disease Tenth Revision (ICD-10) diagnostic codes. All cancer diagnosis must be confirmed via histopathology. The database includes enrollment files, claims data, catastrophic illness files, registry for treatments, and Taiwanese death certificates. Because the information was released strictly for research purposes, the study was exempt from full review by the Internal Review Board of our hospital (No. 160803).

The following items were included in the study: age of diagnosis, sex, surgical method, pathological stage, cell type, histological grade, interval between surgery and adjuvant chemotherapy, 1-year, 3-year, and 5-year survival rates, and median survival time in months. Tumor histology was described according to the World Health Organization classification. All the patients were staged according to the 6^th^ edition of the TNM staging system published in 1997.

### Study sample

This study captured data from the NHIRD collected between January 1, 2004 and December 31, 2010. Using the ICD-10 C34.0, C34.1, C34.2, C34.3, C34.8, and C34.9 diagnostic codes, it was determined that 87,320 patients were diagnosed with lung cancer during this period. Because of missing clinical data, 34,347 patients were excluded from the study and the records of 54,937 patients with complete clinical data were examined. Because adjuvant chemotherapy is recommended for patients with stage IB to IIIA disease after complete surgical resection in the NCCN guidelines, we identified patients with stage IB to IIIA disease in the National Database. Patients with micro or macro residual tumor after surgery were excluded, as were patients with surgical mortality (in-hospital death within 30 days of surgery. Patients who did not receive adjuvant chemotherapy was also excluded. Thus, 1,522 patients with stage IB to IIIA lung cancer who received surgical resection and adjuvant chemotherapy were identified in the NHIRD and included in the analysis. The interval between surgery and chemotherapy was calculated from the date of operation to the date of the first day of adjuvant chemotherapy. The patients were divided into 4 groups according to the interval between surgery and chemotherapy: group 1, < 30 days; group 2, 30–45 days; group 3, 46–60 days; group 4 > 60 days.

### Statistical analysis

SAS software (SAS System for Windows, version 9.2; SAS Institute, Cary, North Carolina) was used to perform the statistical analysis. Overall survival was calculated by the Kaplan-Meier method, and the difference in survival was determined by the log-rank test. Overall survival was calculated based on the time period from the date of surgery to death or December 31, 2012. The date and cause of death were obtained from Taiwanese death certificates, which were updated on December 31, 2012. Continuous data were compared using the 2-tailed *t* test, and comparisons of categorical data were made using the χ^2^ or Fisher exact test. Statistical analysis was considered to be significant with a *p*-value < 0.05.

Univariate and multivariate analyses were performed using the Cox proportional hazards model with SAS software. To investigate the factors influencing overall survival, all of the following clinicopathological factors were included in the multivariate analyses: age, sex, pathological T and N stage, pathological stage, surgical method (pneumonectomy, bilobectomy, lobectomy, wedge resection), cell type, tumor grade, and interval between surgery and chemotherapy.

## Results

Patient basic characteristics and demographic data are summarized in Table 1. The numbers of patients in groups 1, 2, 3, and 4 were 153, 161, 290, and 818, respectively. The 1-year, 3-year and 5-year overall survival rates of each group were shown in Table 2. The 5-year survival rate was 41% in group 1, 48% in group 2, 50% in group 3, and 35% in group 4 (p<0.001). The median survival time was 44.50 months in group 1, 59.53 months in group 2, 67.33 months in group 3 and 36.33 months in group 4.(p<0.001) Overall survival and median survival were significantly different between the 4 groups (*p* < 0.001).

**Table 1:** Clinical demographic data of 1522 lung cancer patients stratified by treatment interval from surgery to chemotherapy.

Characteristic	Group 1	Group 2	Group 3	Group 4	*p*
	< 30 days	0–45 days	45–60 days	> 60 days	
Numbers	153	261	290	818	
Age (years) Median	60.2	59.5	59.5	63.4	<0.0001
Range	25.3–83.3	32.6–84.7	22.5–82.1	31.7–87.4	
Sex (%)					0.0630
Male	105 (68.63)	144 (55.17)	174 (60.00)	488 (59.66)	
Female	48 (31.37)	117 (44.83)	116 (40.00)	330 (40.34)	
Cell type					0.0283
Squamous cell carcinoma	29 (18.95)	49 (18.77)	64 (22.07)	187 (22.86)	
Adenocarcinoma	92 (60.13)	175 (67.05)	182 (62.76)	538 (65.77)	
Small cell	8 (5.23)	7 (2.68)	4 (1.38)	8 (0.98)	
Large cell	3 (1.96)	5 (1.92)	4 (1.38)	12 (1.47)	
Others	21 (13.73)	25 (9.58)	36 (12.41)	73 (8.92)	
Pathological T stage (%)					0.1270
1	18 (11.76)	30 (11.49)	32 (11.03)	70 (8.56)	
2	106 (69.28)	194 (74.33)	205 (70.69)	636 (77.75)	
3	29 (18.95)	37 (14.18)	53 (18.28)	112 (13.69)	
Pathological N stage (%)					<0.0001
0	63 (41.18)	120 (45.98)	127 (43.79)	452 (55.26)	
1	28 (18.3)	49 (18.77)	77 (26.55)	154 (18.83)	
2	62 (40.52)	92 (35.25)	86 (29.66)	212 (25.92)	
Pathological stage (%)					<0.0001
IB	52 (33.99)	102 (39.08)	96 (33.1)	386 (47.19)	
IIA	7 (4.58)	18 (6.9)	31 (10.69)	57 (6.97)	
IIB	24 (15.69)	42 (16.09)	67 (23.1)	145 (17.73)	
IIIA	70 (45.75)	99 (37.93)	96 (33.1)	230 (28.12)	
Histological differentiation					0.0281
Well	13 (8.5)	22 (8.43)	32 (11.03)	96 (11.74)	
Moderately	77 (50.33)	141 (54.02)	156 (53.79)	468 (57.21)	
Poorly	35 (22.88)	67 (25.67)	77 (26.55)	181 (22.13)	
Undifferentiated	3 (1.96)	8 (3.07)	5 (1.72)	10 (1.22)	
Unknown	25 (16.34)	23 (8.81)	20 (6.9)	63 (7.7)	
Surgical method					0.0035
Pneumonectomy	7 (4.58)	13 (4.98)	13 (4.48)	34 (4.16)	
Bilobectomy	3 (1.96)	7 (2.68)	14 (4.83)	28 (3.42)	
Lobectomy	90 (58.82)	174 (66.67)	205 (70.69)	587 (71.76)	
Wedge resection	35 (22.88)	34 (13.03)	27 (9.31)	74 (9.05)	
Other	18 (11.76)	33 (12.65)	31 (10.69)	95 (11.61)	

**Table 2:** Overall survival and median survival of 1522 lung cancer patients stratified by treatment interval from surgery to chemotherapy.

Characteristic	Group 1(n = 153)	Group 2(n = 261)	Group 3(n = 290)	Group 4(n = 818)	p
Overall survival rate					<0.0001
One-year	0.88 (0.82–0.92)	0.88 (0.84–0.92)	0.87 (0.83–0.91)	0.80 (0.77–0.83)	
Three-year	0.57 (0.48–0.64)	0.64 (0.58–0.70)	0.65 (0.58–0.70)	0.50 (0.46–0.53)	
Five-year	0.41 (0.31–0.5)	0.48 (0.41–0.55)	0.50 (0.43–0.57)	0.35 (0.31–0.39)	
Median survival (months)	44.50 (34.70–61.30)	59.53 (44.63–86.06)	67.33 (50.33–76.83)	36.33 (32.17–42.77)	<0.0001

Sex and pathologic T stage were not different between the 4 groups. The mean age at diagnosis of group 4 (63.4 years old) was greater than that of the other groups (*p* < 0.0001). Lobectomies were performed less frequently in group 1 than in the other groups, while wedge resections were performed more frequently in group 1 (*p* = 0.0035). There was no significant difference between the 4 groups in the frequency of pneumonectomies. Group 4 has the greatest number of patients with N0 disease, while group 1 the greatest number with N2. Group 4 had the greatest number of patients with stage IB disease, group 3 has the most stage IIA and IIB disease, and group 4 has the most stage IIIA disease. Tumor cell type and histological grade were significantly different between the 4 group (*p* = 0.0283).

**Fig 1:**
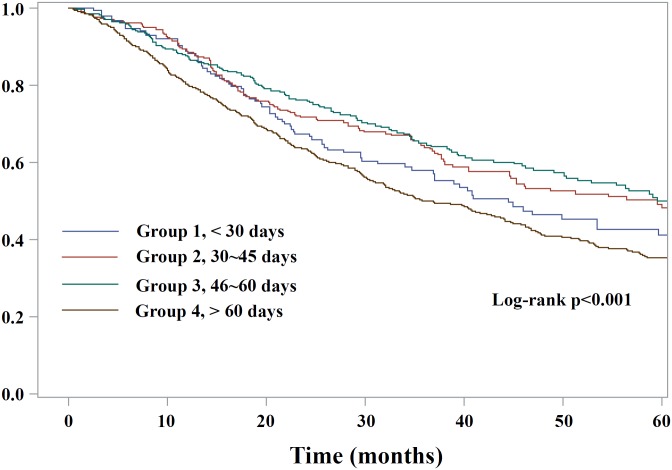
Kaplan-Meier survival curves for lung cancer patient who underwent complete surgical treatment and adjuvant chemotherapy. The survival difference was significant between group 4 and other groups (*p* < 0.001). Patients receiving adjuvant chemotherapy more than 60 days after surgery had a worse survival rate.

Kaplan-Meier survival curves stratified by treatment interval was shown in Fig 1. Group 3 has the best survival rate, while group 4 the worst survival. Patients receiving adjuvant chemotherapy more than 60 days after surgery had a worse survival rate (*p* < 0.001).

Univariate analysis indicated that age of diagnosis, sex, pathological T stage, pathological N stage, surgical method, cell type, and tumor grade were significantly associated with overall survival (Table 3). In multivariate analysis, age, sex, pathological T stage, pathological N stage, surgical method, cell type, and interval between surgery and chemotherapy were independent risk factors for survival. Older age at diagnosis, male sex, advanced pathological stage, surgical methods of pneumonectomy and wedge resection, small cell cancer type, and interval between surgery and first course of chemotherapy more than 60 days had an adverse effect on overall survival (Table 3).

**Table 3:** Univariate and multivariate analysis.

	Univariate		Multivariate	
	HR (95%CI)	*p*	HR (95% CI)	*p*
Age	1.018 (1.011–1.025)	<0.0001	1.011 (1.004–1.019)	0.0024
Sex				
Male	1.645 (1.421–1.905)	<0.0001	1.456 (1.241–1.707)	<0.0001
Female (ref)	1	-	1	-
T				
1 (ref)	1	-	1	-
2	1.058 (0.834–1.342)	0.6412	1.167 (0.905–1.504)	0.2350
3	1.400 (1.057–1.854)	0.0191	1.417 (1.05–1.912)	0.0225
N				
0 (ref)	1	-	1	-
1	1.121 (0.931–1.349)	0.2278	1.251 (1.031–1.518)	0.0234
2	1.397 (1.194–1.634)	<0.0001	1.463 (1.238–1.73)	<0.0001
Surgical method				
Pneumonectomy	1.699 (1.257–2.297)	0.0006	1.472 (1.07–2.026)	0.0176
Bilobectomy	1.388 (0.961–2.005)	0.0809	1.147 (0.78–1.687)	0.4856
Lobectomy (ref)	1	-	1	-
Wedge resection	1.580 (1.292–1.932)	<0.0001	1.538 (1.251–1.891)	<0.0001
Other	1.423 (0.919–2.201)	0.1134	1.509 (0.966–2.359)	0.0706
Cell type				
Squamous cell	1.379 (1.165–1.632)	0.0002	1.013 (0.835–1.228)	0.8987
Adenocarcinoma (ref)	1	-	1	-
Small cell	2.476 (1.582–3.875)	<0.0001	2.126 (1.318–3.427)	0.0020
Large cell	0.887 (0.488–1.612)	0.6935	0.657 (0.345–1.254)	0.2031
Others	1.487 (1.195–1.85)	0.0004	1.429 (1.137–1.797)	0.0022
Differentiation				
Well (ref)	1	-	1	-
Moderately	1.151 (0.901–1.471)	0.2606	1.056 (0.824–1.353)	0.6690
Poorly	1.314 (1.006–1.716)	0.0451	1.119 (0.848–1.477)	0.4256
Undifferentiated	1.380 (0.78–2.443)	0.2688	1.392 (0.754–2.571)	0.2904
Unknown	1.342 (0.98–1.838)	0.067	0.988 (0.71–1.376)	0.9432
Interval surgery to chemo				
< 30 days (ref)	1	-	1	-
30–45 days	0.831 (0.627–1.099)	0.1945	0.937 (0.706–1.244)	0.6549
45–60 days	0.785 (0.595–1.035)	0.0859	0.873(0.658–1.158)	0.3456
> 60 days	1.243 (0.982–1.572)	0.0700	1.437 (1.127–1.831)	0.0034

CI, confidence interval; HR, hazard ratio.

## Discussion

This study investigated the clinical impact of the interval between surgery and chemotherapy in patients with lung cancer using data from the Taiwanese NHIRD 2006 to 2012, and included 1,522 cases. The results indicated that patients receiving adjuvant chemotherapy more than 60 days after surgery had a significantly lower 5-year survival rate than in all of the groups of patients receiving chemotherapy earlier.

According to the latest NCCN guideline for management of non-small cell lung cancer, patients with stage IB-IIIA disease who undergo complete surgical resection should receive adjuvant chemotherapy. Although the optimal timing to administer adjuvant chemotherapy in patients with lung cancer has not been addressed in the literature, it has been examined in other cancer types. In breast cancer, several studies have discussed early or delayed adjuvant chemotherapy but have used different cutoff times. Alkis et al. [11] set the cutoff time at 45 days, Pronzato et al. [12] set it at 35 days, and Colleoni et al. [13] set it at 21 days. In spite of different cutoff times, the conclusions were similar; earlier administration was associated with significant survival benefit as compared to delayed administration. Lohrisch et al. [14] divided breast cancer patients into 4 groups with chemotherapy administration at less than 4 weeks, 4 to 8 weeks, 8 to 12 weeks, and 12 to 24 weeks after surgery and found that the 8 to 12 week group had the best disease-free survival rate and the 12 to 24 week group the worst disease free survival rate.

Numerous studies have examined the timing of adjuvant chemotherapy in colorectal cancer. A systematic review and meta-analysis in 2011 by Biagi et al. [15] identified 10 eligible studies involving 15,410 patients, and found that a 4 week increase in time to adjuvant chemotherapy was associated with a significant decrease in both overall survival and disease-free survival. The results suggested adjuvant chemotherapy should not be administered later than 12 weeks after surgery. Lima et al. [16] and Yu et al. [17] also reported results suggesting that adjuvant chemotherapy should not be administered later than 12 weeks. Czaykowski et al. [18] reported that adjuvant chemotherapy should be given within 8 weeks after surgery. On the other hand, Peixoto et al. [19] reported that a delay of oxaliplatin-based adjuvant chemotherapy beyond 8 weeks did not appear to be associated with inferior outcomes. Overall, however, most studies in colorectal cancer support that adjuvant chemotherapy should not be delayed.

With respect to gastric cancer, the first study published in 2015 by Park et al. [20] found that adjuvant chemotherapy administered more than 8 weeks after surgery was associated with worse survival outcomes.

We have reported the first analysis regarding the timing of adjuvant chemotherapy after complete surgical resection in lung cancer patients, and found that patients receiving adjuvant chemotherapy more than 60 days after surgery had a worse survival rate. The interval between surgery and adjuvant chemotherapy was still an independent prognostic factor in multivariate analysis. A possible theory for these findings is that gross complete surgical resection might not entirely eradicate cancer cells. Adjuvant chemotherapy is aimed at eliminating cancer cells and suppressing the growth of hidden micrometastasis. The later that adjuvant chemotherapy is administered, the greater the opportunity for micrometastasis to proliferate. On the basis of this hypothesis, adjuvant chemotherapy should begin as soon as possible after surgery. Delayed chemotherapy may increase the potential risk of tumor regrowth. If the patient could recovered well from surgery, chemotherapy should be administrated within 60 days. On the other hand, patients for whom adjuvant chemotherapy is delayed may have had surgical complications or be in a poorer physical condition, and thus may have a diminished response to adjuvant chemotherapy.

The strength of our analysis is large and nationwide sample size, and examining an issue that has not been previously researched. Several limitations still exists, however, including the retrospective analysis and lack of data with respect to the cause of death, chemotherapy regimen, and complications of surgery and chemotherapy.

In conclusion, patients with lung cancer who undergo curative resection have poorer survival outcomes when adjuvant chemotherapy is delayed more than 60 days after surgery. These results suggest that adjuvant chemotherapy should be administered within 60 days after surgical resection.
